# Enhanced prognostic value of a composite nutritional-inflammatory index (P-CONUT) for predicting mortality risk in patients initiating peritoneal dialysis

**DOI:** 10.1371/journal.pone.0323318

**Published:** 2025-05-22

**Authors:** Xing Li, Xueli Zhou, Kuo Li, Li Pu, Xueqin He, Xia Liu, Hui Zhong, Dengyan Ma

**Affiliations:** 1 Department of Nephrology, West China Hospital of Sichuan University, Chengdu, Sichuan, China; 2 West China School of Nursing, Sichuan University, Chengdu, Sichuan, China; Osaka University of Pharmaceutical Sciences, JAPAN

## Abstract

**Background and Objectives:**

Nutritional and inflammatory indicators are key to predicting outcomes in peritoneal dialysis (PD) patients. This study evaluates the prognostic value of the Prognostic Nutritional Index (PNI), Controlling Nutritional Status (CONUT) score, and a novel composite score, P-CONUT, which integrates both nutritional and inflammatory status, to improve risk prediction and management in PD patients.

**Methods:**

This retrospective study included 810 PD patients. The primary outcome was all-cause mortality. Kaplan-Meier survival curves compared outcomes across groups, and log-rank tests assessed differences. Univariate and multivariate cox regression analyses identified independent mortality predictors. The prognostic performance of CONUT, PNI, and P-CONUT was evaluated using the area under the curve (AUC) and integrated AUC comparisons. Net reclassification improvement (NRI) and integrated discrimination improvement (IDI) were used to assess the incremental value of P-CONUT over CONUT and PNI. Decision curve analysis (DCA) assessed the clinical utility of the models. A nomogram incorporating significant predictors was developed to aid in prognosis prediction.

**Results:**

Cox regression identified P-CONUT as an independent predictor of all-cause mortality (G2 vs. G1: HR = 0.354, 95% CI: 0.238–0.528, p < 0.001; G3 vs. G1: HR = 0.385, 95% CI: 0.270–0.549, p < 0.001). P-CONUT demonstrated superior prognostic performance (AUC = 0.790, 95% CI: 0.751–0.829), with improvements over CONUT (AUC = 0.611, 95% CI: 0.561–0.661) and PNI (AUC = 0.636, 95% CI: 0.587–0.686). The NRI for P-CONUT over CONUT and PNI was 0.331 (95% CI: 0.156–0.408) and 0.357 (95% CI: 0.221–0.428), respectively. The IDI for P-CONUT compared to CONUT and PNI was 0.111 (95% CI: 0.011–0.145) and 0.112 (95% CI: 0.018–0.149), respectively. DCA demonstrated that P-CONUT provided a greater net benefit than both CONUT and PNI across a range of risk thresholds.

**Conclusion:**

P-CONUT is a strong independent predictor of mortality in PD patients, outperforming both CONUT and PNI in prognostic accuracy. The composite P-CONUT score, integrating both nutritional and inflammatory status, provides superior predictive value, aiding in more precise risk stratification. This score, coupled with other significant prognostic factors, offers a reliable tool for improving the long-term management and clinical decision-making for PD patients.

## Introduction

Peritoneal dialysis (PD) is an essential renal replacement therapy for patients with end-stage renal disease (ESRD), with around 11% of ESRD patients worldwide receiving PD, particularly in areas with limited access to hemodialysis [1,2]. PD offers advantages such as greater patient autonomy and a lower risk of cardiovascular complications compared to hemodialysis [3]. However, despite these benefits, the long-term survival rate for PD patients remains suboptimal, with 5-year survival rates ranging from 40% to 70% [4,5]. Several factors hinder long-term survival in PD patients, with malnutrition and chronic inflammation being particularly significant [6]. Both of these conditions are strongly linked to increased mortality.

Studies have shown that between 25% and 40% of PD patients suffer from malnutrition, with these individuals facing a two- to three-fold increased risk of mortality compared to those with normal nutritional status [7,8]. In addition to malnutrition, chronic low-grade inflammation is a common and persistent issue in PD patients. This inflammation contributes to complications such as cardiovascular disease, infections, and poor prognosis [9,10]. The coexistence and interrelationship between protein-energy malnutrition and chronic low-grade inflammation in PD patients further exacerbate the risk of mortality in this population [11]. Therefore, a comprehensive and accurate early assessment of nutritional and inflammatory status, aimed at screening and identifying high-risk individuals, is crucial for optimizing interventions and improving prognostic management.

To assess these factors, several tools have been developed. Among them, the controlled nutritional risk (CONUT) score and the prognostic nutritional index (PNI) are widely used [12]. The CONUT score evaluates nutritional status based on serum albumin, total cholesterol, and lymphocyte count, while the PNI combines serum albumin and peripheral blood lymphocyte count to assess immune function and inflammation levels [13,14]. Despite considering similar serum-based markers, the prognostic effects of CONUT score and PNI in PD patients show some inconsistent results, and a more reliable indicator is needed for widespread adoption of these nutritional indicators.

In recent years, the Nutritional-Inflammatory Complex Score (NICS) method has emerged as a promising tool for prognostic evaluation by combining nutritional and inflammatory markers to assess patient outcomes [15,16]. Building on this concept, our study introduces the P-CONUT score, a composite measure that integrates the CONUT and PNI scores, specifically designed for PD patients. This study aims to evaluate the prognostic value of the P-CONUT score, particularly in predicting all-cause mortality in PD patients, through a retrospective analysis.

In conclusion, this study seeks to enhance the predictive accuracy of mortality risk in PD patients by introducing the P-CONUT score, a novel composite measure that integrates nutritional and inflammatory markers. By focusing on both nutritional and inflammatory status, this score provides a more comprehensive tool for risk stratification in PD patients, addressing the limitations of existing scoring systems. The findings of this study could contribute to improved clinical decision-making, enabling early identification of high-risk individuals and more targeted interventions, ultimately aiming to improve long-term survival and quality of life for PD patients.

## Materials and methods

### 1. Study population and design

This retrospective observational cohort study was conducted at West China Hospital, Sichuan University. We enrolled patients who had initial peritoneal dialysis (PD) between June 2, 2010, and December 31, 2017. These patients were monitored from the initiation of dialysis until June 30, 2023, or their last recorded clinical visit. Exclusion criteria included patients under 18 years of age, those diagnosed with breast, lung, gastrointestinal, or hematologic cancers, and those with incomplete data. After applying these criteria, a total of 810 patients were included in the final analysis.

The study was conducted in accordance with the ethical principles outlined in the Declaration of Helsinki and its subsequent amendments. Approval was obtained from the Biomedical Ethics Review Committee of West China Hospital, Sichuan University (protocol code: 2019–793), and all participants provided written informed consent. The authors did not have access to identifiable information about individual participants before or after data collection.

### 2. Data collection and measurements

#### 2.1. Data collection.

Clinical and laboratory data were collected from individual medical records at the initiation of dialysis. These included demographic information (age, sex), the cause of end-stage renal disease (ESRD), systolic and diastolic blood pressure, hemoglobin, uric acid, total cholesterol, LDL/HDL cholesterol, triglycerides, glutamic oxaloacetic transaminase (AST), alanine transaminase (ALT), total protein, blood glucose, blood urea, serum potassium, calcium, albumin, total lymphocyte count, and globulin levels. Blood tests were conducted to calculate the CONUT score and PNI within one month prior to the initiation of dialysis.

### 2.2. Calculations of CONUT score, PNI and P-CONUT

The CONUT score was calculated based on serum albumin (g/dL), total lymphocyte count (count/mm³), and total cholesterol (mg/dL), as detailed in Supplementary S1 Table. The PNI was calculated using the following formula: 10 × serum albumin (g/dL) + 0.005 × total peripheral lymphocyte count (µL). Cutoff values for both CONUT and PNI were determined using the X-tile program, a tool designed to identify optimal cutoff points through statistical tests. The results are presented in S1 Fig. The program evaluated all possible divisions of the marker data, calculating associations at each division. The log-rank test was used for survival analysis, and the mean test assessed relationships with other markers. Optimal cutoff values were identified by maximizing the chi-squared value across potential divisions, enabling the stratification of patients into low or high CONUT and PNI groups.

“P-CONUT” was defined by integrating the CONUT score and PNI, based on previously established criteria. Patients were classified into three P-CONUT groups: G1 (high CONUT [5–12] and low PNI [≤38.6]), G2 (low CONUT [0–4] and high PNI [>38.6]), and G3 (high CONUT [5–12] and high PNI [>38.6]). Notably, no patients were classified as low CONUT (0–4) and low PNI (≤38.6).

### 2.3. Follow up

Follow-up was conducted through phone interviews and a review of medical records, with the study concluding in June 2023. Patients who transferred from West China Hospital were censored at their last known contact date. For overall survival (OS) analysis, patients who were still alive as of June 30, 2023, were censored. OS was defined as the time from dialysis initiation to death from any cause. The primary endpoint of the study was all-cause mortality. Follow-up continued for all patients until either June 2023 or the time of death.

### 3. Statistical analyses

Statistical analyses were performed using SPSS version 27.0 and R version 4.4.0. The Kolmogorov-Smirnov test was used to assess the normality of continuous variables. Normally distributed data are presented as mean ± standard deviation, while non-normally distributed data are expressed as median (interquartile range). Continuous variables were compared using the Independent Samples t-test or the Mann-Whitney U test, as appropriate. Categorical variables were analyzed using the chi-square test or Fisher’s exact test.

Kaplan-Meier survival curves were generated to estimate overall survival (OS) rates, with differences assessed using the log-rank test. Cox proportional hazards regression models were used to identify independent predictors of all-cause mortality. Variables with p-values < 0.05 in univariate analysis were included in multivariable models, which were constructed using backward stepwise selection to identify independent risk factors for OS. Given potential interactions among the CONUT score, PNI, and P-CONUT, three separate multivariable models were developed, each including one of these variables.

The predictive performance of CONUT, PNI, and P-CONUT for OS was compared using the integrated area under the curve (iAUC) to assess time-dependent discriminatory ability. Additionally, net reclassification improvement (NRI) and integrated discrimination improvement (IDI) were used to evaluate the incremental predictive value of P-CONUT over CONUT and PNI.

Decision Curve Analysis (DCA) was performed to assess the clinical utility of CONUT, PNI, and P-CONUT. DCA compares the net benefit (NB) of using each model at various risk thresholds, considering the trade-off between true positives and false positives. NB was calculated across a range of threshold probabilities, with higher net benefits indicating better clinical decision-making performance. DCA provides insight into the clinical value of each model by evaluating its impact on patient outcomes.

A p-value < 0.05 was considered statistically significant. A nomogram was constructed based on significant prognostic factors, including P-CONUT, to aid in predicting PD patient outcomes.

## Results

### 1. Clinical characteristics

The median follow-up period was 9.05 years (range, 5.52–13.08 years). A total of 810 patients were included (see Supplementary S2 Fig). Patient characteristics are summarized in Table 1. Based on CONUT score classification, 252 patients (31.1%) were in the low-CONUT group and 558 (68.9%) into the high-CONUT group. For the PNI scores, 212 patients (26.2%) were classified into the low-PNI group, while 598 patients (73.8%) were classified into the high-PNI group. Detailed characteristics of the patients across the CONUT and PNI groups are provided in Tables 2 and 3.

**Table 1:** Demographic and baseline characteristic of peritoneal dialysis patients (n = 810).

Characteristic	Value
Age (years), mean±SD	50.4114.40
Male gender (%)	489(60.3%)
Body mass index (kg/m^2^)	22.45(20.16,24.91)
Cause of ESRD	
Glomerulonephritis (%)	521(64.3%)
Hypertension (%)	105(12.9%)
Diabetes mellitus (%)	114(14.0%)
Other/unknown (%)	70(8.5%)
Systolic blood pressure (mmHg)	146.9821.50
Diastolic blood pressure (mmHg)	89.00(79.00,100.00)
Hemoglobin (g/L)	81.00(71.00,95.00)
Uric acid (umol/L)	469.38153.69
Total cholesterol (mmol/L)	71.73(60.48,85.01)
LDL cholesterol (mmol/L)	2.19(1.67,2.77)
HDL cholesterol (mmol/L)	1.18(0.91,1.45)
Triglycerides (mmol/L)	1.39(0.99,1.89)
Aspartate aminotransferase (U/L)	16.00(10.00,25.00)
Alanine aminotransferase (U/L)	19.00(14.00,26.00)
Total protein (g/L)	62.70(57.10,68.40)
Albumin-to-globulin ratio	1.46(1.25,1.68)
Blood glucose (mmol/L)	5.02(4.57,5.74)
Blood urea (mmol/L)	27.90(20.21,35.74)
Serum potassium (mmol/L)	4.45(4.01,4.97)
Serum calcium (mmol/L)	2.04(1.86,2.16)
CONUT score	5.00(4.00,7.00)
PNI score	42.826.46

Note: SD standard deviation, ESRD end-stage renal disease, LDL low density lipoprotein, HDL high density lipoprotein, CONUT controlling nutritional status, PNI prognostic nutritional index.

**Table 2:** Characteristic in patients with different CONUT score.

Characteristic	CONUT		*P* value
**Low-CONUT** **(≤4), (n = 252)**	**High-CONUT** **(>4), (n = 558)**	
Age (years), mean±SD	50.2314.92	50.5514.13	0.804
Male gender, n(%)	153(60.7%)	336(60.2%)	0.893
Body mass index (kg/m^2^), median (inter-quartile range)	23.38(20.91,25.50)	22.20(19.99,24.55)	0.154
Cause of ESRD			
Glomerulonephritis, n(%)	169(67.1%)	352(63.1%)	0.256
Diabetes mellitus, n(%)	24(9.5%)	90(16.1%)	0.027
Hypertension, n(%)	41(32.1%)	75(13.4%)	0.282
Other/unknown, n(%)	17(6.7%)	40(7.2%)	0.828
Systolic blood pressure (mmHg), mean±SD	141.8722.36	149.2820.74	<0.001
Diastolic blood pressure (mmHg), Median (inter-quartile range)	87.00(77.00,99.00)	89.00(80.00,100.00)	0.042
Hemoglobin (g/L), median (inter-quartile range)	89.00(77.00,101.00)	78.00(68.00,90.50)	<0.001
Uric acid (umol/L), mean±SD	492.04152.99	458.83152.99	0.006
Total cholesterol (mmol/L), median (inter-quartile range)	76.23(65.70,94.05)	69.30(58.05,81.45)	<0.001
LDL cholesterol (mmol/L), median (inter-quartile range)	2.51(1.86,3.22)	2.08(1.63,2.60)	<0.001
HDL cholesterol (mmol/L), median (inter-quartile range)	1.17(0.91,1.46)	1.18(0.91,1.44)	0.546
Triglycerides (mmol/L), median (inter-quartile range)	1.64(1.09,2.16)	1.30(0.96,1.74)	<0.001
Aspartate aminotransferase (U/L), median (inter-quartile range)	16.00(11.00,24.00)	16.00(10.00,25.50)	0.580
Alanine aminotransferase (U/L), median (inter-quartile range)	18.00(14.00,25.00)	19.00(14.00,26.00)	0.660
Total protein (g/L), median (inter-quartile range)	67.05(63.15,71.10)	60.30(54.90,66.15)	<0.001
Albumin-to-globulin ratio, median (inter-quartile range)	1.52(1.34,1.73)	1.44(1.22,1.65)	<0.001
Blood glucose (mmol/L), median (inter-quartile range)	5.03(4.56,5.74)	5.02(4.57,5.75)	0.852
Blood urea (mmol/L), median (inter-quartile range)	27.78(21.62,33.97)	28.03(19.37,36.30)	0.772
Serum potassium (mmol/L), median (inter-quartile range)	4.54(4.17,5.08)	4.40(3.99,4.91)	0.011
Serum calcium (mmol/L), median (inter-quartile range)	2.11(1.95,2.20)	2.01(1.81,2.13)	<0.001

Note: SD standard deviation, ESRD end-stage renal disease, LDL low density lipoprotein, HDL high density lipoprotein, CONUT controlling nutritional status.

**Table 3:** Characteristic in patients with different PNI score.

Characteristic	PNI		*P* value
**Low-PNI** **(≤38.6), (n = 212)**	**High-PNI** **(>38.6), (n = 589)**	
Age (years), mean±SD	51.56 ± 14.12	50.01 ± 14.48	0.178
Male gender, n(%)	134(27.4%)	355(72.6%)	0.326
Body mass index (kg/m^2^), median (inter-quartile range)	22.48(20.13,24.77)	22.45(20.20,24.97)	0.665
Cause of ESRD			
Glomerulonephritis, n(%)	124(23.8%)	397(76.2%)	0.034
Diabetes mellitus, n(%)	46(40.4%)	68(59.6%)	<0.001
Hypertension, n(%)	25(21.6%)	91(78.4%)	0.215
Other/unknown, n(%)	17(29.8%)	40(70.2%)	0.515
Systolic blood pressure (mmHg), mean±SD	153.85 ± 20.59	144.54 ± 21.31	<0.001
Diastolic blood pressure (mmHg), median (inter-quartile range)	89.50(80.00,101.00)	88.50(79.00,99.00)	0.196
Hemoglobin (g/L), median (inter-quartile range)	73.00(66.00,83.00)	86.00(73.75,97.25)	<0.001
Uric acid (umol/L), mean±SD	424.27 ± 149.51	485.38 ± 152.09	<0.001
Total cholesterol (mmol/L), median (inter-quartile range)	69.12(56.93,84.33)	72.18(61.11,85.23)	0.054
LDL cholesterol ((mmol/L), median (inter-quartile range)	2.11(1.60,2.75)	2.23(1.70,2.78)	0.122
HDL cholesterol ((mmol/L), median (inter-quartile range)	1.07(0.86,1.38)	1.22(0.94,1.49)	0.002
Triglycerides ((mmol/L), median (inter-quartile range)	1.39(1.00,1.85)	1.38(0.98,1.90)	0.777
Aspartate aminotransferase (U/L), median (inter-quartile range)	17.00(10.00,27.00)	15.00(10.00,24.00)	0.424
Alanine aminotransferase (U/L), median (inter-quartile range)	20.00(15.00,29.00)	18.00(14.00,24.50)	0.001
Total protein (g/L), median (inter-quartile range)	54.40(50.30,58.80)	65.20(60.35,69.70)	<0.001
Albumin-to-globulin ratio, median (inter-quartile range)	1.26(1.09,1.48)	1.52(1.34,1.74)	<0.001
Blood glucose (mmol/L), median (inter-quartile range)	5.07(4.57,5.82)	5.00(4.57,5.73)	0.499
Blood urea (mmol/L), median (inter-quartile range)	25.17(16.45,34.15)	28.45(21.60,36.23)	0.002
Serum potassium (mmol/L), median (inter-quartile range)	4.28(3.88,4.72)	4.52(4.10,5.07)	<0.001
Serum calcium (mmol/L), median (inter-quartile range)	1.95(1.77,2.07)	2.08(1.91,2.19)	<0.001

Note: SD standard deviation, ESRD end-stage renal disease, LDL low density lipoprotein, HDL high density lipoprotein, PNI prognostic nutritional index.

### 2 Kaplan–Meier survival curves according to the CONUT, PNI, and P-CONUT

**Fig 1:**
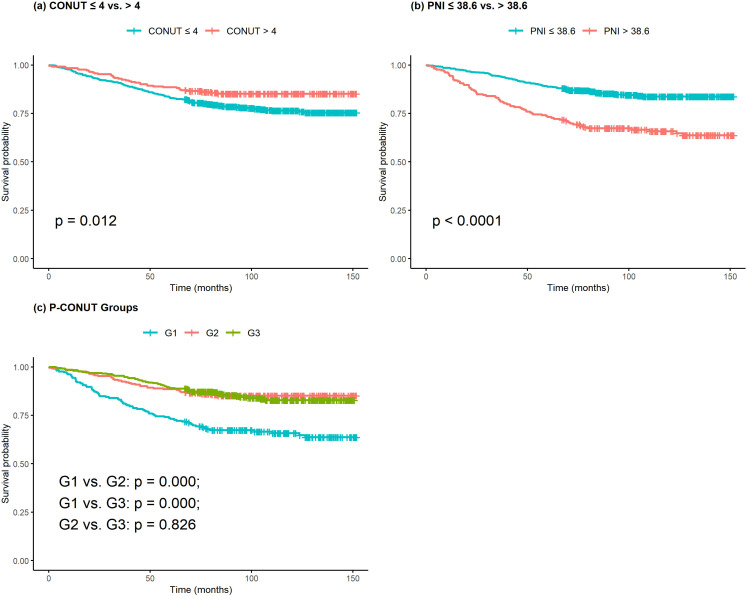
Kaplan–Meier survival curves based on the CONUT scores, PNI, and P-CONUT. Figure 1a shows the Kaplan-Meier survival curve according to CONUT. Figure 1b shows the Kaplan-Meier survival curves based on the PNI. Figure 1c shows the Kaplan-Meier survival curve according to P-CONUT.

Kaplan–Meier survival curves for CONUT, PNI, and P-CONUT are shown in Fig 1. For CONUT (Fig 1a), the 5-year overall survival (OS) rates were 88.5% for the low-CONUT group and 83.0% for the high-CONUT group (p = 0.012). For PNI (Fig 1b), the 5-year OS rates were 73.1% for the low-PNI group and 88.8% for the high-PNI group (p < 0.001). The P-CONUT survival curves (Fig 1c) demonstrated significant differences, with 5-year OS rates of 73.1%, 88.1%, and 89.0% for groups G1, G2, and G3, respectively (p < 0.001).

### 3. Cox regression modeling process

**Fig 2:**
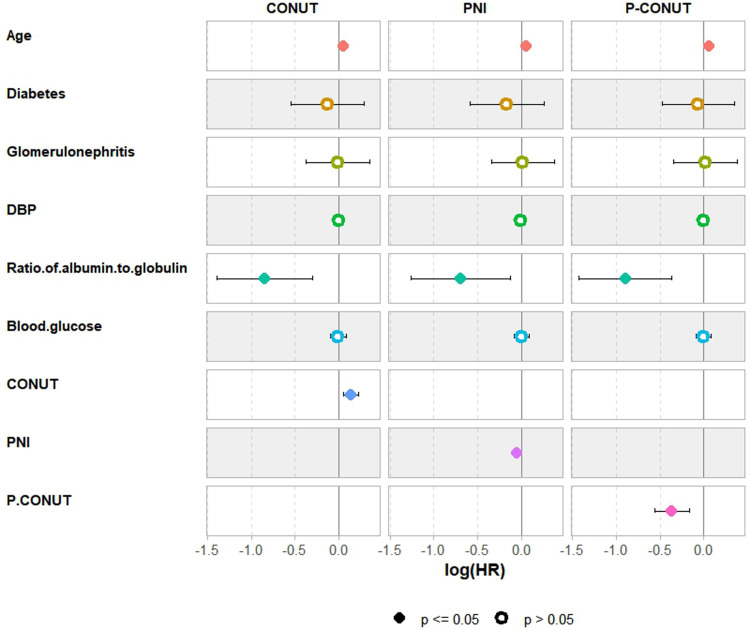
Cox regression forest plots associated with overall survival (n ** = 810).** The figure presents forest plots of Cox regression models assessing factors associated with overall survival in peritoneal dialysis (PD) patients. Three models are shown: Model 1 (CONUT), Model 2 (PNI), and Model 3 (P-CONUT). Each plot displays the beta coefficients with corresponding confidence intervals for variables including age, diabetes, glomerulonephritis, diastolic blood pressure, albumin-to-globulin ratio, blood glucose, and nutritional-inflammatory indices (CONUT, PNI, and P-CONUT). Colored dots represent individual variables, with black dots indicating statistically significant associations (p ≤ 0.05) and open circles denoting non-significant results (p > 0.05). Negative beta values suggest a protective effect, while positive beta values indicate an increased risk of mortality.

Univariable analysis identified several factors significantly associated with OS, including age (p < 0.001), diabetes (p < 0.001), glomerulonephritis (p = 0.001), diastolic blood pressure (p < 0.001), albumin-to-globulin ratio (p < 0.001), and blood glucose (p = 0.027). CONUT, PNI, and P-CONUT scores were also significant prognostic indicators, with G2 vs. G1 and G3 vs. G1 groups showing strong significance (both p < 0.001) (Table 4). After multivariable adjustment using backward selection, independent predictors of OS included age, albumin-to-globulin ratio, CONUT (HR = 1.478, 95% CI: 1.012–2.159, p = 0.043), PNI (HR = 2.688, 95% CI: 1.972–3.666, p < 0.001), and P-CONUT [G2 vs. G1: HR = 0.354, 95% CI: 0.238–0.528, p < 0.001; G3 vs. G1: HR = 0.385, 95% CI: 0.270–0.549, p < 0.001] (Table 5, Fig 2). Although diabetes did not reach statistical significance, it was included in the model due to its clinical relevance in predicting mortality risk.

**Table 4:** Univariable analysis associated with the overall survival (n = 810).

Characteristics		Univariable analysis	
		HR (95% CI)	P value
Age (years)	<70	Ref.	
	≥70	4.742(3.403,6.608)	＜0.001
Gender (n)	female	Ref.	
	male	0.845(0.621,1.151)	0.286
Body mass index (kg/m^2^)	<25	Ref.	0.746
	≥25	0.890(0.615,1.288)	0.537
Diabetes (n)	no	Ref.	
	yes	2.286(1.617,3.231)	＜0.001
Glomerulonephritis (n)	no	Ref.	
	yes	1.713(1.257,2.333)	＜0.001
Hypertension (n)	no	Ref.	
	yes	1.086(0.686,1.718)	0.725
Systolic BP (mmHg)	≤140	Ref.	
	>140	1.227(0.887,1.696)	0.217
Diastolic BP (mmHg)	≤90	Ref.	
	>90	1.964(1.408,2.738)	0.000
Hemoglobin (g/L)	<115	Ref.	
	≥115	0.607(0.225,1.636)	0.324
Uric acid (umol/L)	≤380	1.159(0.828,1.622)	0.390
	>380	Ref.	
Total cholesterol (mmol/L)	≤5.7	1.026(0.570,1.847)	0.931
	>5.7	Ref.	
LDL cholesterol (mmol/L)	≤4.0	0.792(0.389,1.611)	0.519
	>4.0	Ref.	
HDL cholesterol (mmol/L)	≤0.9	1.191(0.841,1.686)	0.326
	>0.9	Ref.	
Triglycerides (mmol/L)	≤1.83	1.174(0.819,1.682)	0.382
	>1.83	Ref.	
Aspartate aminotransferase (U/L)	<40	1.179(0.704,1.975)	0.532
	≥40	Ref.	
Alanine aminotransferase (U/L)	<35	0.718(0.462,1.115)	0.140
	≥35	Ref.	
Total protein (g/L)	<65.0	Ref.	
	≥65.0	1.044(0.763,1.427)	0.789
Ratio of albumin to globulin	<1.2	2.569(1.860,3.548)	＜0.001
	≥1.2	Ref.	
Blood glucose (mmol/L)	≤5.9	Ref.	
	>5.9	1.467(1.044,2.063)	0.027
Blood urea (mmol/L)	≤8.8	Ref.	
	>8.8	0.887(0.393,2.005)	0.774
Serum potassium (mmol/L)	≤5.3	Ref.	
	>5.3	0.880(0.562,1.379)	0.577
Blood calcium (mmol/L)	<2.11	1.329(0.953,1.853)	0.094
	≥2.11	Ref.	
CONUT	≤4	Ref.	
	>4	1.597(1.107,2.303)	0.012
PNI	≤38.6	2.541(1.867,3.458)	<0.001
	>38.6	Ref.	
P-CONUT	G1	Ref.	
	G2	0.383(0.258,0.569)	<0.001
	G3	0.401(0.282,0.570)	<0.001

**Table 5:** Multivariable analysis associated with the overall survival (n = 810).

Characteristics	Model 1 (CONUT)	Model 2 (PNI)	Model 3 (P-CONUT)
HR (95% CI)	HR (95% CI)	HR (95% CI)
Age	1.058(1.044,1.072)^*^	1.059(1.045,1.073)^*^	1.057(1.043,1.070)^*^
Diabetes	0.879(0.577,1.337)	0.848(0.557,1.290)	0.938(0.619,1.422)
Glomerulonephritis	0.987(0.687,1.418)	1.018(0.709,1.463)	1.017(0.707,1.462)
Diastolic BP	0.994(0.982,1.006)	0.994(0.982,1.006)	0.994(0.983,1.006)
Ratio of albumin to globulin	0.430(0.249,0.741)^*^	0.499(0.282,0.885)^*^	0.409(0.240,0.695)^*^
Blood glucose	0.991(0.907,1.083)	0.999(0.917,1.090)	0.996(0.912,1.088)
CONUT	1.148(1.058,1.247)^*^	–	–
PNI	–	0.950(0.924,0.977)^*^	–
P-CONUT	–	–	0.694(0.569,0.846)^*^

Note:

*p < 0.05

### 4. Comparison of integrated AUC and Incremental predictive value of P-CONUT

**Fig 3:**
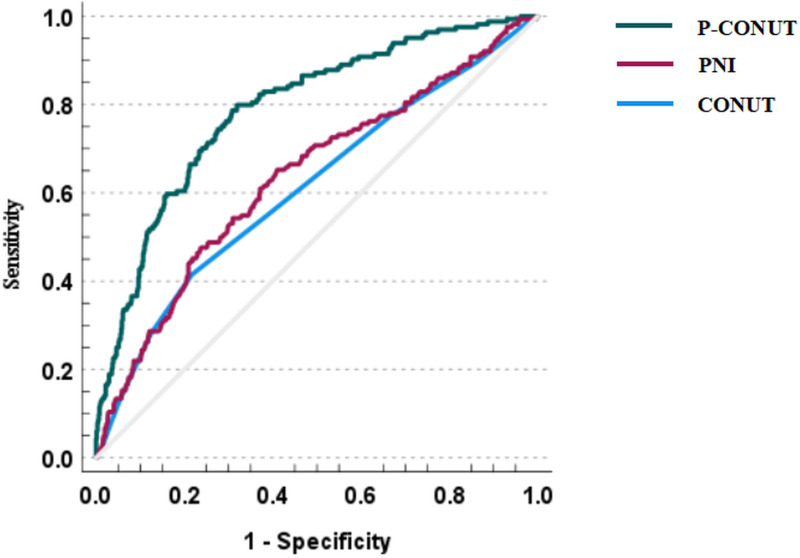
The time-dependent receiver operating characteristic curve of P-CONUT, CONUT score and PNI. This figure presents the time-dependent ROC curves for P-CONUT (green line), PNI (red line), and CONUT (blue line), illustrating their ability to predict overall survival in peritoneal dialysis (PD) patients. The x-axis represents 1 - specificity, and the y-axis represents sensitivity. The diagonal gray line represents the reference line (AUC = 0.5), indicating no discriminatory ability. P-CONUT demonstrates the highest area under the curve (AUC), suggesting superior prognostic performance compared to PNI and CONUT.

The AUC for CONUT in predicting all-cause mortality was 0.611 (95% CI: 0.561–0.661), with a sensitivity of 41.5% and specificity of 78.3%. The AUC for PNI was 0.636 (95% CI: 0.587–0.686), with a sensitivity of 65.2% and specificity of 59.0%. The P-CONUT model demonstrated the highest predictive performance, with an AUC of 0.790 (95% CI: 0.751–0.829), sensitivity of 79.9%, and specificity of 68.1%. The time-dependent ROC curve for P-CONUT outperformed both CONUT (bootstrap iAUC mean difference = 0.000; 95% CI: 0.127–0.231) and PNI (bootstrap iAUC mean difference = 0.000; 95% CI: 0.103–0.205) over the follow-up period (Fig 3).

The enhancement effect of P-CONUT was further evaluated using the NRI and IDI. Compared to the CONUT score, the NRI value for P-CONUT was 0.331 (95% CI: 0.156–0.408) and the IDI value was 0.111 (95% CI: 0.011–0.145). Compared to the PNI score, the NRI for P-CONUT was 0.357 (95% CI: 0.221–0.428) and the IDI was 0.112 (95% CI: 0.018–0.149) (Table 6). These results suggest that the P-CONUT model has superior ability in predicting survival outcomes in PD patients.

**Table 6:** Comparison of NRI and IDI among three models.

Index	NRI		IDI	
Estimate	95%CI	Estimate	95%CI
P-CONUT vs. CONUT	0.331^*^	[0.156-0.408]^*^	0.111	[0.011-0.145]^*^
P-CONUT vs. PNI	0.357^*^	[0.221-0.428]^*^	0.112	[0.018-0.149]^*^

Note:

*p < 0.05

### 5. DCA for CONUT, PNI, and P-CONUT

**Fig 4:**
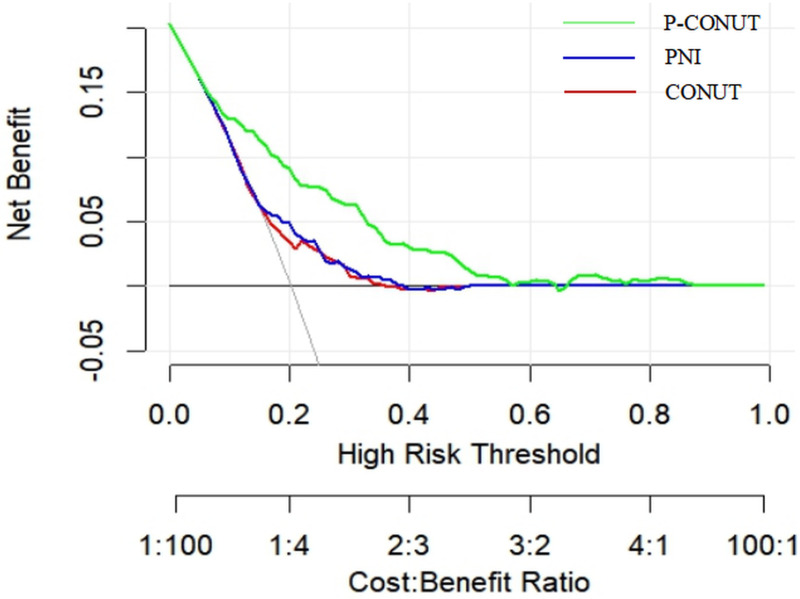
Decision Curve Analysis (DCA) Comparing the Clinical Utility of P-CONUT, CONUT, and PNI for Predicting Mortality in Peritoneal Dialysis Patients. This figure presents the decision curve analysis (DCA) comparing the net clinical benefit of P-CONUT (green line), PNI (blue line), and CONUT (red line) in predicting mortality risk in peritoneal dialysis (PD) patients. The x-axis represents the high-risk threshold, while the y-axis represents the net benefit. The analysis demonstrates that P-CONUT consistently provides a higher net benefit across a wide range of risk thresholds compared to PNI and CONUT, suggesting its superior clinical utility in risk stratification and decision-making.

DCA was conducted to assess the clinical utility of the CONUT, PNI, and P-CONUT scores (Fig 4). The results demonstrated that, for patients with a high-risk threshold ranging from 9% to 66%, P-CONUT consistently provided a greater net benefit (NB) compared to the strategies of treating all patients or treating none. Notably, P-CONUT, as a composite score integrating both CONUT and PNI, outperformed both individual scores (CONUT and PNI) across all risk thresholds, highlighting its superior prognostic value for predicting mortality risk in PD patients.

### 6. Predictive nomogram for P-CONUT

**Fig 5:**
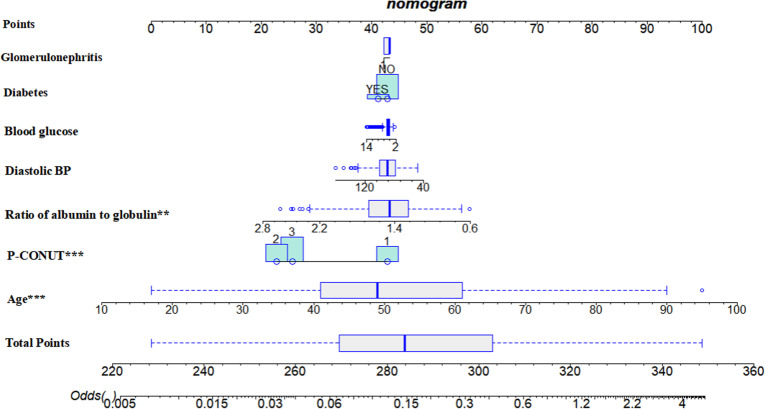
Nomogram for Predicting Prognosis in Initial Dialysis Patients with Chronic Kidney Disease Based on P-CONUT and Clinical Variables. This nomogram was developed to predict prognosis in peritoneal dialysis (PD) patients using P-CONUT and other significant clinical variables. The included predictors are glomerulonephritis, diabetes, blood glucose, diastolic blood pressure, albumin-to-globulin ratio, P-CONUT, and age. Each variable is assigned a corresponding score on the Points scale, and the total score is calculated by summing the individual scores. The total points correspond to the predicted odds of adverse outcomes, as indicated on the Odds scale at the bottom. The nomogram provides a visual tool for individualized risk assessment, facilitating clinical decision-making and early intervention strategies in PD patients.

A nomogram was constructed using significant prognostic factors for predicting adverse outcomes in PD patients (Fig 5). The included predictors were glomerulonephritis, diabetes, blood glucose, diastolic blood pressure, albumin-to-globulin ratio, P-CONUT, and age. Each predictor was assigned a score on the nomogram scale, and the total score correlated with the predicted odds of adverse outcomes. Glomerulonephritis and diabetes were binary variables, while blood glucose, diastolic blood pressure, and albumin-to-globulin ratio were continuous variables. Lower P-CONUT values reflected better nutritional status. Age was a significant risk factor, with older patients having a higher risk. The total score provided an estimate of the likelihood of an adverse event.

## Discussion

This study aimed to evaluate and compare the prognostic value of three nutritional-inflammatory indicators—CONUT, PNI, and P-CONUT—in predicting all-cause mortality in PD patients. The mortality risk in PD patients is strongly influenced by their nutritional and inflammatory status. While CONUT and PNI are widely used in clinical practice, their individual predictive accuracy is limited. To address this limitation, we developed the P-CONUT score, a composite index that integrates CONUT and PNI to provide a more comprehensive assessment of nutritional and inflammatory status. Our findings demonstrated that P-CONUT outperformed CONUT and PNI, with significantly higher AUC, NRI, and IDI values. Furthermore, the development of a nomogram based on P-CONUT offers an effective clinical tool for individualized risk stratification and decision-making.

Previous studies have underscored the importance of nutritional and inflammatory status in predicting outcomes among PD patients [17–19]. The CONUT score, which incorporates serum albumin, total lymphocyte count, and cholesterol levels, has been associated with adverse outcomes in both CKD and PD populations [20]. Similarly, the PNI, derived from serum albumin and lymphocyte count, reflects inflammatory status and has been reported to predict survival in PD patients [21,22]. In our study, the PNI demonstrated a stronger association with overall survival than the CONUT score, aligning with prior research that identified PNI as a critical prognostic factor in PD patients [23]. However, our results stand in contrast to the findings of Yang Y et al., who reported that the CONUT score was a better predictor of outcomes than PNI in PD patients [24]. The discrepancy between our results and theirs may be attributable to several factors, including differences in the cutoff values used for each variable, variations in sample sizes, and the inclusion of patients at different stages of disease progression [25,26]. These differences underscore the need for further research to explore how these factors impact the predictive value of nutritional indices in PD patients.

While both CONUT and PNI provide valuable prognostic information, they independently assess either nutritional depletion or inflammatory response but fail to comprehensively integrate both aspects. The P-CONUT score addresses this gap by combining the strengths of CONUT and PNI into a unified index, offering a more holistic assessment of MICS.

Statistical evaluation of P-CONUT confirmed its robustness. For instance, P-CONUT achieved an AUC of 0.790 (95% CI: 0.751–0.829), significantly higher than CONUT (0.611; 95% CI: 0.561–0.661) and PNI (0.636; 95% CI: 0.587–0.686).These findings are consistent with previous studies showing that composite scores combining nutritional and inflammatory parameters, such as the comprehensive systemic inflammatory response index and nutritional parameters such as albumin and height, are superior to individual markers in predicting patient outcomes [27,28]. However, the P-CONUT score uniquely integrates CONUT and PNI, offering a tailored tool specifically for PD populations. Alongside superior AUC values, P-CONUT showed significant improvements in NRI and IDI, indicating better reclassification and discrimination of mortality risk compared to CONUT and PNI. For example, the NRI for P-CONUT relative to CONUT was 0.331 (95% CI: 0.156–0.408) and 0.357 (95% CI: 0.221–0.428) relative to PNI, while the corresponding IDI values were 0.111 (95% CI: 0.011–0.145) and 0.112 (95% CI: 0.018–0.149), respectively.

In this study, high P-CONUT scores were strongly associated with increased mortality risk, underscoring its potential clinical value. By capturing both nutritional depletion and inflammatory response, P-CONUT provides a more holistic assessment of patients’ overall health status, which is particularly relevant in PD care. Patients with high P-CONUT scores demonstrated worse 5-year survival rates compared to those with lower scores, highlighting its ability to stratify patients based on mortality risk. DCA further demonstrated that P-CONUT provided greater net clinical benefit across a range of risk thresholds, emphasizing its practical applicability in optimizing treatment decisions.

The clinical utility of P-CONUT lies in its ability to guide targeted interventions. High-risk patients identified by P-CONUT could benefit from early nutritional support, such as dietary adjustments or supplementation, and strategies to reduce inflammation, including optimizing dialysis prescriptions or using anti-inflammatory therapies [29–31]. Moreover, P-CONUT can inform broader care strategies, such as adjusting dialysis modalities, managing comorbidities, or increasing the frequency of patient monitoring [32,33]. These interventions could lead to improved survival outcomes and better resource allocation in clinical practice.

To support clinical application, we developed a nomogram that incorporates the P-CONUT score along with other significant prognostic factors, such as age, diabetes, and blood pressure. This nomogram provides a user-friendly tool for risk assessment, enabling clinicians to predict patient outcomes and customize treatment plans accordingly. By integrating evidence-based data into clinical decision-making, the nomogram enhances communication with patients and their families, supporting discussions on prognosis and treatment options. This approach ensures that care plans are tailored to align with individual patient values and preferences.

Despite its promising findings, this study has several limitations. First, this was a single-center retrospective study, and the patients in our study were generally younger and had fewer coexisting conditions, such as diabetes, which may limit the generalizability of our findings to other patient populations. Second, we only evaluated patient data at the initiation of dialysis and did not analyze how changes in nutritional status post-dialysis might affect prognosis. Future prospective studies are needed to assess the longitudinal impact of PNI and CONUT scores on patient outcomes. Lastly, while P-CONUT demonstrated improved predictive accuracy, further research is required to compare its utility with other emerging prognostic tools and validate its implementation in diverse clinical settings.

## Conclusions

In conclusion, this study highlights the critical role of nutritional and inflammatory status in predicting mortality risk among PD patients. The P-CONUT score, as a composite indicator of these factors, provides superior prognostic value compared to individual markers, offering a practical tool for risk stratification and personalized care. By integrating P-CONUT into clinical workflows, healthcare providers can better identify high-risk patients, implement timely interventions, and ultimately improve long-term outcomes for PD populations.

## Supporting information

S1 Fig Defining optimal cut-off value for PNI and CONUT using X-tile program. (A) PNI: X-tile plots (left) and histogram (right) demonstrating the optimal cutoff value for Prognostic Nutritional Index (PNI). The left panel visualizes the population distribution, where green and red areas represent high and low subpopulations, respectively. The right panel displays the frequency distribution of PNI scores, with the optimal cutoff point marked.(B) CONUT: Similar X-tile plots (left) and histogram (right) illustrating the optimal cutoff determination for Controlling Nutritional Status (CONUT) score. The left panel shows the stratification of patients into low- and high-risk groups, while the right panel presents the corresponding frequency distribution. These cutoff values were determined to maximize prognostic discrimination for survival analysis in peritoneal dialysis patients.
(TIF)

S2 Fig Flowchart of patient selection for the study cohort. The diagram illustrates the inclusion and exclusion process for peritoneal dialysis (PD) patients enrolled in the study. A total of 861 patients initiated PD between June 2010 and December 2017. After excluding 30 patients who transferred to other PD centers or were lost to follow-up and 14 patients diagnosed with tumors, 817 patients remained. Subsequently, 7 patients without available CONUT or PNI data were further excluded, resulting in a final study cohort of 810 patients included in the analysis.
(TIF)

S1 Table CONUT scoring system. The Controlling Nutritional Status (CONUT) score is calculated based on serum albumin level, total cholesterol level, and total lymphocyte count. Each component is assigned a score according to predefined ranges. The total CONUT score ranges from 0 to 12 and reflects the degree of nutritional impairment: 0–1 = normal, 2–4 = mild, 5–8 = moderate, and 9–12 = severe malnutrition.
(DOCX)
