# Supplementary figures and images for: Enhanced prognostic value of a composite nutritional-inflammatory index (P-CONUT) for predicting mortality risk in patients initiating peritoneal dialysis
Source: PLoS One. 2025 May 22;20(5):e0323318. doi: 10.1371/journal.pone.0323318 (PMC12097555; doi:10.1371/journal.pone.0323318)

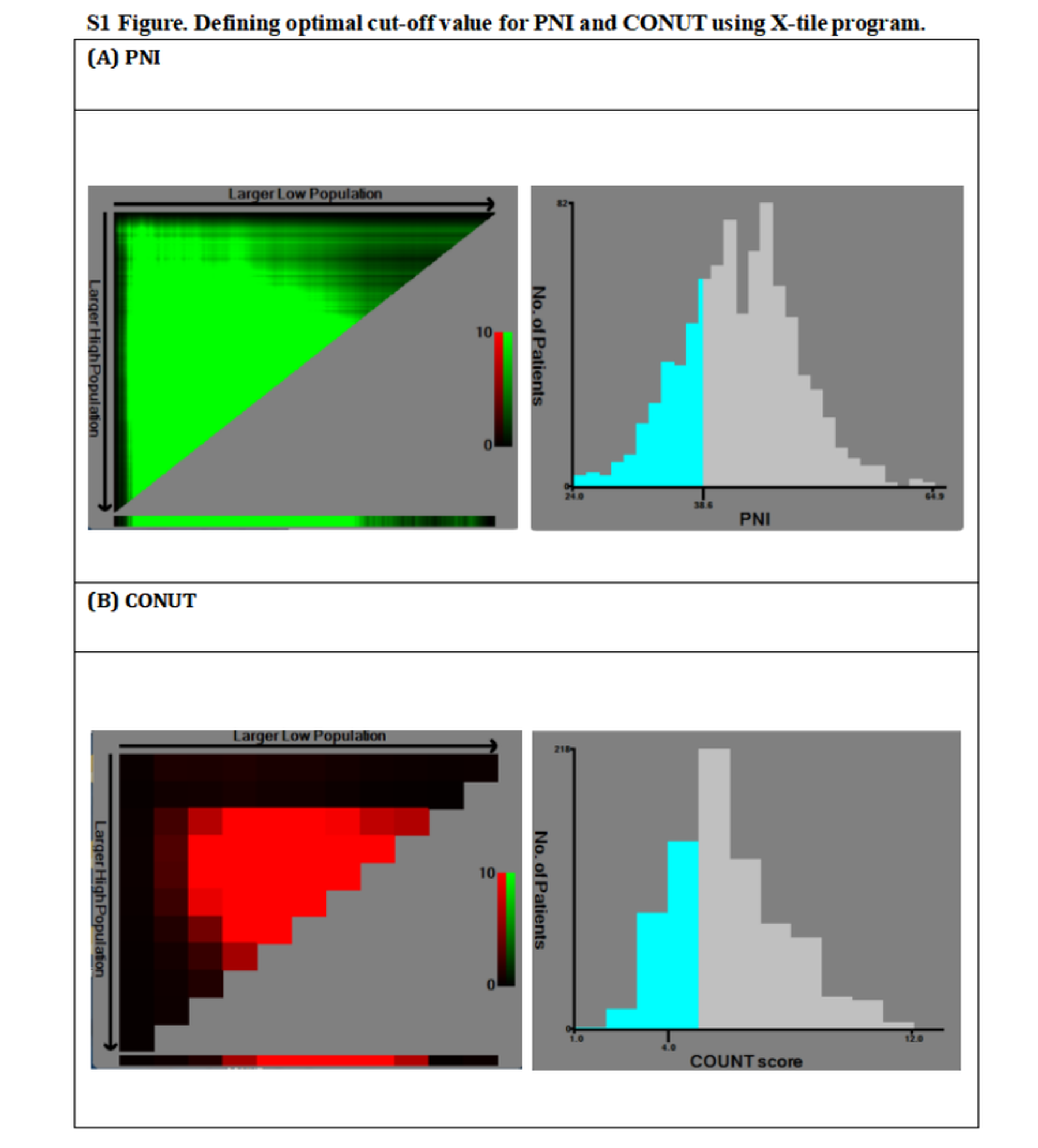

Supplement: S1 Fig — (A) PNI: X-tile plots (left) and histogram (right) demonstrating the optimal cutoff value for Prognostic Nutritional Index (PNI). The left panel visualizes the population distribution, where green and red areas represent high and low subpopulations, respectively. The right panel displays the frequency distribution of PNI scores, with the optimal cutoff point marked.(B) CONUT: Similar X-tile plots (left) and histogram (right) illustrating the optimal cutoff determination for Controlling Nutritional Status (CONUT) score. The left panel shows the stratification of patients into low- and high-risk groups, while the right panel presents the corresponding frequency distribution. These cutoff values were determined to maximize prognostic discrimination for survival analysis in peritoneal dialysis patients. (TIF) [file pone.0323318.s001.tif]

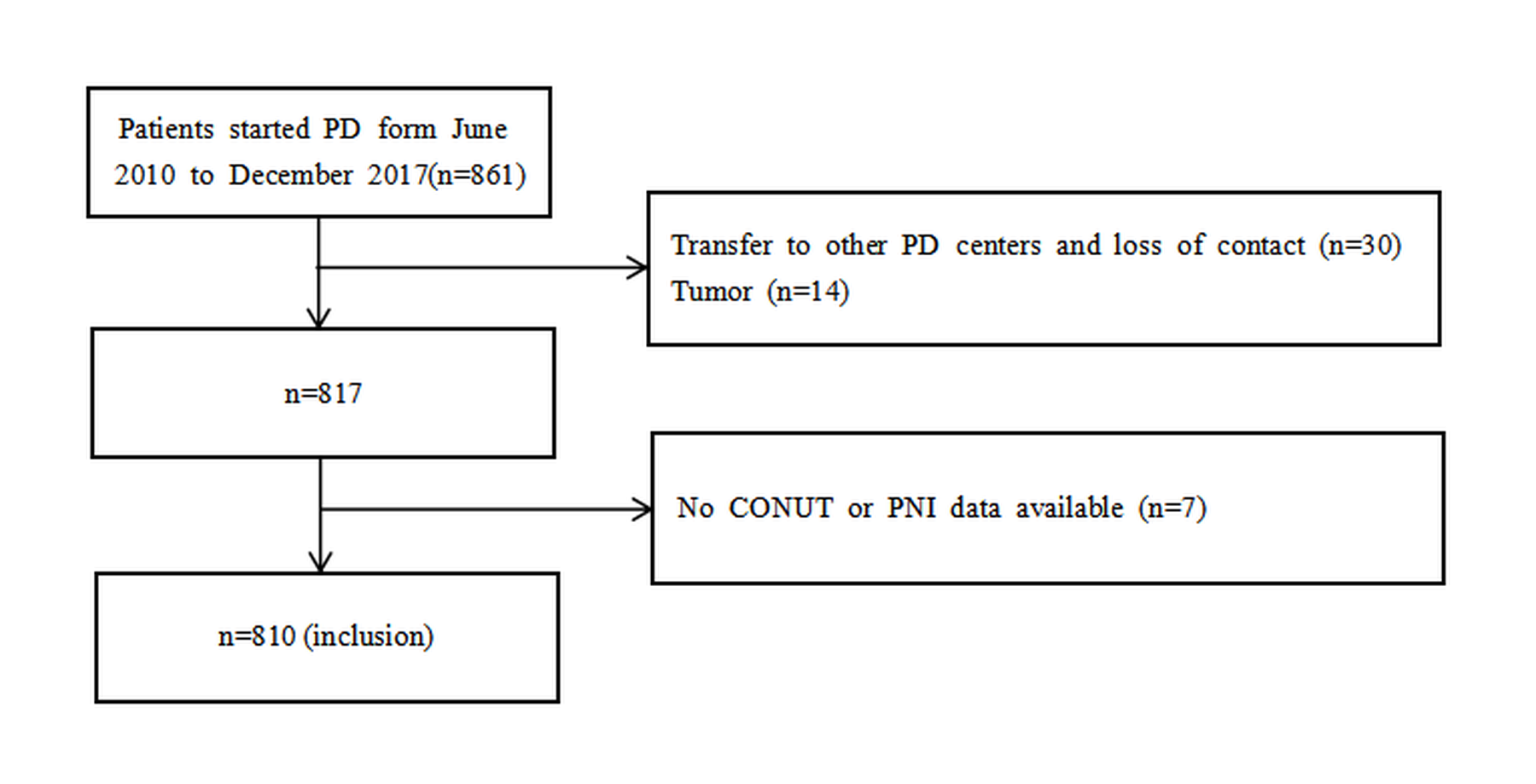

Supplement: S2 Fig — The diagram illustrates the inclusion and exclusion process for peritoneal dialysis (PD) patients enrolled in the study. A total of 861 patients initiated PD between June 2010 and December 2017. After excluding 30 patients who transferred to other PD centers or were lost to follow-up and 14 patients diagnosed with tumors, 817 patients remained. Subsequently, 7 patients without available CONUT or PNI data were further excluded, resulting in a final study cohort of 810 patients included in the analysis. (TIF) [file pone.0323318.s002.tif]
