# Supplementary material for: Enhanced prognostic value of a composite nutritional-inflammatory index (P-CONUT) for predicting mortality risk in patients initiating peritoneal dialysis
Source: PLoS One. 2025 May 22;20(5):e0323318. doi: 10.1371/journal.pone.0323318 (PMC12097555; doi:10.1371/journal.pone.0323318)
Supplement: S1 Table — The Controlling Nutritional Status (CONUT) score is calculated based on serum albumin level, total cholesterol level, and total lymphocyte count. Each component is assigned a score according to predefined ranges. The total CONUT score ranges from 0 to 12 and reflects the degree of nutritional impairment: 0–1 = normal, 2–4 = mild, 5–8 = moderate, and 9–12 = severe malnutrition. (DOCX) [file pone.0323318.s003.docx]

**S1 Table. CONUT score.**

| Variables | Range | Score |
| --- | --- | --- |
| Serum albumin (g/dL) | ≥3.50 | 0 |
|  | 3.00-3.49 | 2 |
|  | 2.50-2.99 | 4 |
|  | ＜2.50 | 6 |
| Cholesterol (mg/dL) | ≥180 | 0 |
|  | 140-179 | 1 |
|  | 100-139 | 2 |
|  | ＜100 | 3 |
| Lymphocyte count (mm^3^) | ≥1600 | 0 |
|  | 1200-1599 | 1 |
|  | 800-1199 | 2 |
|  | ＜800 | 3 |
| Total score | 0-1 | Normal |
|  | 2-4 | Mild |
|  | 5-8 | Moderate |
|  | 9-12 | Severe |
